# The Impact of Perceived Social Power and Dangerous Context on Social Attention

**DOI:** 10.1371/journal.pone.0114077

**Published:** 2014-12-02

**Authors:** Gege Cui, Shen Zhang, Haiyan Geng

**Affiliations:** 1 Department of Psychology, Peking University, Beijing, China; 2 Department of Psychology, University of Wisconsin-Whitewater, Whitewater, Wisconsin, United States of America; Ecole Normale Supérieure, France

## Abstract

Past research has shown that position in a social hierarchy modulates one's social attention, as in the gaze cueing effect. While studies have manipulated the social status of others with whom the participants interact, we believe that a sense of one's own social power is also a crucial factor affecting gaze following. In two experiments, we primed the social power of participants, using different approaches, to investigate the participants' performance in a subsequent gaze cueing task. The results of Experiment 1 showed a stronger gaze cueing effect among participants who were primed with low social power, compared to those primed with high social power. Our predicted gender difference (i.e., women showing a stronger gaze cueing effect than men) was confirmed and this effect was found to be dominated by the lower social power condition. Experiment 2 manipulated the level of danger in the context and replicated the joint impact of gender and one's perceived social power on gaze cueing effect, especially in the low danger context, in comparison to the high danger context. These findings demonstrate that one's perceived social power has a concerted effect on social attention evoked by gaze, along with other factors such as gender and characteristics of the environment, and suggest the importance of further research on the complex relationship between an individual's position in the social hierarchy and social attention.

## Introduction

Social hierarchy is ubiquitous in human society, and is a fundamental aspect of people's interactions [1], [2]. Being able to recognize who ranks higher in the social hierarchy is critical for survival, as those of higher status occupy more resources and may provide information or protection to others [3]. A recent study showed a bias towards high social status in social attention, where participants automatically distributed their attention based on the social status of others, and paid more attention to those of higher social status than to those of medium or low status [4].

Our study focuses on social attention evoked by gaze, which is also modified by social hierarchy. It has been found that humans tend to follow the gaze direction of others; they rapidly shift attention to where others are gazing [5] and recognize a target more quickly and accurately when the target appears in those locations, compared to when the target appears in a location opposite to the gaze direction [6], [7], [8]. This phenomenon is known as the gaze cueing effect, or gaze-induced joint attention. It exists even when participants are told that the gaze direction is irrelevant or opposite to the location of the target [9], [10], [11], or when the gaze cue is subliminal [12]. Gaze contains a willingness to communicate and signals an object of interest [13]. The sensitivity to gaze develops among infants who are as young as three months old [14], and the ability of gaze following is found across species from humans to other mammals such as monkeys and dogs [15], [16], [17]. Gaze induced social attention is also subject to the impact of social factors such as social status. For example, in an animal study, greater gaze cueing effect was found among dominant macaque monkeys when they were presented with an image of the face of a high-status monkey, compared to that of a low-status monkey; whereas submissive monkeys followed the gaze direction of other monkeys, regardless of their social status [18]. Similarly, a stronger gaze cueing effect was found among human participants when they were presented with the image of a more dominant face, compared to a less dominant face [19], which is in keeping with the finding that facial dominance positively predicts one's social status [20]. Direct evidence has also been obtained from research in which participants adjusted their behavior in response to different social statuses of two faces presented to them, and showed a stronger gaze cueing effect in response to the face of an individual described as having a higher status in the curriculum vitae that the participants had read previously [21], and the effect persisted with a very short presentation of faces such as 50 ms [22]. In another study [23], participants' racial group membership were found to affect their gaze cueing: while members of the majority group oriented their attention in response to gaze cues provided by peers but not by members of the minority group, members of the minority group oriented their attention for both [23]. This racial group effect on gaze cueing may reflect the effect of social status, as the majority group normally possesses higher social status.

Since social status is a relative characteristic perceived during interaction, in the studies described above, seeing a high (low) status face is likely to make observers feel that they are at a relatively lower (higher) position in the social hierarchy, and have less (more) control over other individuals or resources. In other words, interacting with such faces can elicit experiences of less (more) social power among participants [2]. Therefore, when previous research manipulated the social status of another person (the object of social interaction), the modulation effect of social status on gaze-induced joint attention may be accounted for by: 1) perceiving another's social status: people are willing to follow the gaze of those who have a high status; or 2) perceiving one's own social power: people with low social power are more sensitive to gaze cues, and thus, are more likely to follow another's gaze. In fact, evidence suggests that, with greater social power, people show less perspective-taking and have less consideration for the thoughts and feelings of others. For example, when primed with high social power, participants were less likely to draw the letter “E” on their forehead in the orientation as seen from an observer's perspective, compared to those primed with low social power [24]. In addition, with less social power, people conformed more to peer pressure, and were more influenced by foreign examples in their imaginary drawings [25]. Nevertheless, the role of one's perceived social power in more fundamental processes, such as social attention, has not been addressed.

We believe that examining the perception of one's own social power is important to fully understand how social status affects a basic process like gaze-following behavior during social interactions. In reality, individuals do not always know the social status of those with whom they interact. Therefore, it would also be ecologically valid to explore whether or not and how the perceived social power of oneself modulates gaze-following behavior. In Experiment 1, we primed the participants' perception of their social power (high vs. low) by asking them to recall a past experience related to different levels of social power [26], [27], while controlling for the face that the participants interacted with. This experiment is the first to focus on the effect of one's own perceived social power on his/her social attention.

An important moderator of the gaze cueing effect is the context of the interaction. For example, the gaze cueing effect is stronger for fearful faces, compared to neutral faces [28], [29], it may because a fearful expression often implies a dangerous context [30]. Past research, however, has not consistently found a changed gaze cueing effect toward faces with different emotional expressions [31], [32], again, likely due to the context. For example, participants showed a stronger gaze cueing effect for fearful faces, relative to happy faces, only if the context itself was threatening [33], [34], [35]. These findings indicate that the gaze cueing effect may only be moderated when the level of threat or danger in the context is “sufficient.”

Our Experiment 2 aims at investigating whether or not a dangerous context moderates the gaze cueing effect, while participants are primed with high or low senses of social power. In this regard, the only study we have found so far manipulated the social status of the other with whom participants interact. Specifically, after participants viewed non-threatening pictures, such as smiling babies and scenes of nature that are rated as high in terms of pleasure and low for arousal, the gaze cueing effect was found for both more and less dominant faces. Nevertheless, after participants viewed threatening pictures, such as attacks and accidents that are rated as low in terms of pleasure and high for arousal, only the more dominant faces produced the gaze cueing effect [36]. We want to examine whether or not the priming of participants' social power has an effect that is similar to that in the earlier research. More importantly, given that the level of threat or danger might affect the size of the gaze cueing effect, we manipulated the degree of danger in the context by including both low and high levels of danger. Specifically, we primed participants to imagine hiking out of the mountains as a low danger context, and escaping from an earthquake as a high danger context. We believe this manipulation is particularly suitable for addressing our research question regarding different levels of dangerous context. Considering that China has witnessed severe earthquakes, and the mass media still spreads earthquake-related information, such as survival guides, the recent real life context and vivid memories would make our priming task of the earthquake a more dangerous context than the mountain hiking situation, or other imagined situations used in previous research [25]. At the same time, we assigned participants a role of being either a leader or a member of a team, which has been shown to effectively prime social power [26]. Therefore, Experiment 2 primed the participants' high or low social power as well as their perception for different levels of dangerous context, and explored whether these two factors jointly modulate the gaze cueing effect.

Since the findings from previous research on social status and the gaze cueing effect could be explained by individuals of relatively less power being sensitive to the social gaze cue, in our study, we also considered the gender factor. Compared to men, women have lower status positions [37], [38], and they also show greater social sensitivity in social contexts [39], as well as present a stronger gaze cueing effect [40], [41]. We speculate that the greater social sensitivity maybe due to women being lower in social status or lack of power. If so, given past research suggests that women's greater social sensitivity may explain the gender difference in the gaze cueing effect [39], temporarily priming different levels of social power should affect the performance of women in the gaze cueing task. We hypothesized that, in relative to priming with low social power, priming high social power will reduce women's gaze cueing effect, as well as the gender difference on this effect.

In summary, we extended past research on social status and the gaze following behavior by priming the social power of participants and examining its interactions with gender and context. Specifically, Experiment 1 primed one's perception of social power at different levels and Experiment 2 further manipulated the dangerous contexts to explore the possible modulation of perceived social power and context on gaze-induced joint attention, and how gender plays a role in these effects.

## Methods

### Experiment 1

#### Participants

Sixty undergraduate students of Peking University (28 men, 32 women; Mean age  = 22.4 years, SD = 2.8 years) participated in Experiment 1 and received monetary compensation for their time. Eight participants did not follow the instructions when completing the initial priming task on social power (see the Procedure section) and were therefore excluded. Data analysis was conducted on the remaining 52 participants who completed the study as required.

#### Ethics statement

The ethics review committee of the Department of Psychology, Peking University approved the protocol details of our study, including the purpose, procedure, and materials. Participants provided written consent before taking part in this experiment and were fully debriefed at the end of the study.

#### Materials

The program for the gaze cueing task was generated by Matlab 7. All stimuli were presented on a 17-inch ViewSonic Professional Series P220f+ CRT monitor (1024×768 at 100 Hz) against a black background (RGB: 0, 0, 0).

Specifically, the faces were created with FaceGen 3.4 (Copyright 2009, Singular Inversions Inc.) and presented at the center of the screen with a 3.5×3.5 visual angle. To ensure that the faces were not familiar or relevant to participants, we used a young, typical eastern Asian face with neutral gender characteristics and neutral emotional expression as the prototype. The prototype was modified in its direction of gaze to form different face stimuli. Three types of faces were used in total: 1) face with direct gaze (the face was gazing straight ahead); 2) face with averted gaze to the left (at an angle of 54°); and 3) face with averted gaze to the right (at an angle of 54°).

The fixation point was a white cross (RGB: 255, 255, 255) extending 0.5°×0.5° of the visual angle. The target stimulus was a white dot (RGB: 255, 255, 255) at 0.1°×0.1° of the visual angle, located at a 7° visual angle horizontally away from the center of the computer screen.

#### Procedure

Individual participants were asked to complete a priming task followed by a gaze cueing task. In the priming task, participants were randomly assigned to either a high or a low social power priming condition (26 participants, including 13 men and 13 women, were assigned to each condition). They were asked to write an anonymous essay in detail about their past experience in 10 minutes, in which they controlled, managed, and affected others (high social power priming), or were controlled, managed, and affected by others (low social power priming). Participants were also told that this task was for a different study and was not related to the subsequent task.

The participants then completed a gaze cueing task right after the priming task, with their heads supported by a chin rest at a viewing distance of 45 cm from the computer screen. The gaze cueing task began with eight practice trials, and was followed by two, 60-formal-trial blocks with a 30 s interval between the blocks. The practice trials were used to familiarize participants with the gaze cueing task, and the results were not recorded. Each formal trial began with the fixation point being presented for 900 ms at the center of the computer screen, on which participants were instructed to focus their attention. A face with a direct gaze was then presented for 600 ms, and replaced with a face with an averted gaze. The target appeared at the 200 ms SOA with the presentation of the face with an averted gaze.

**Figure 1:**
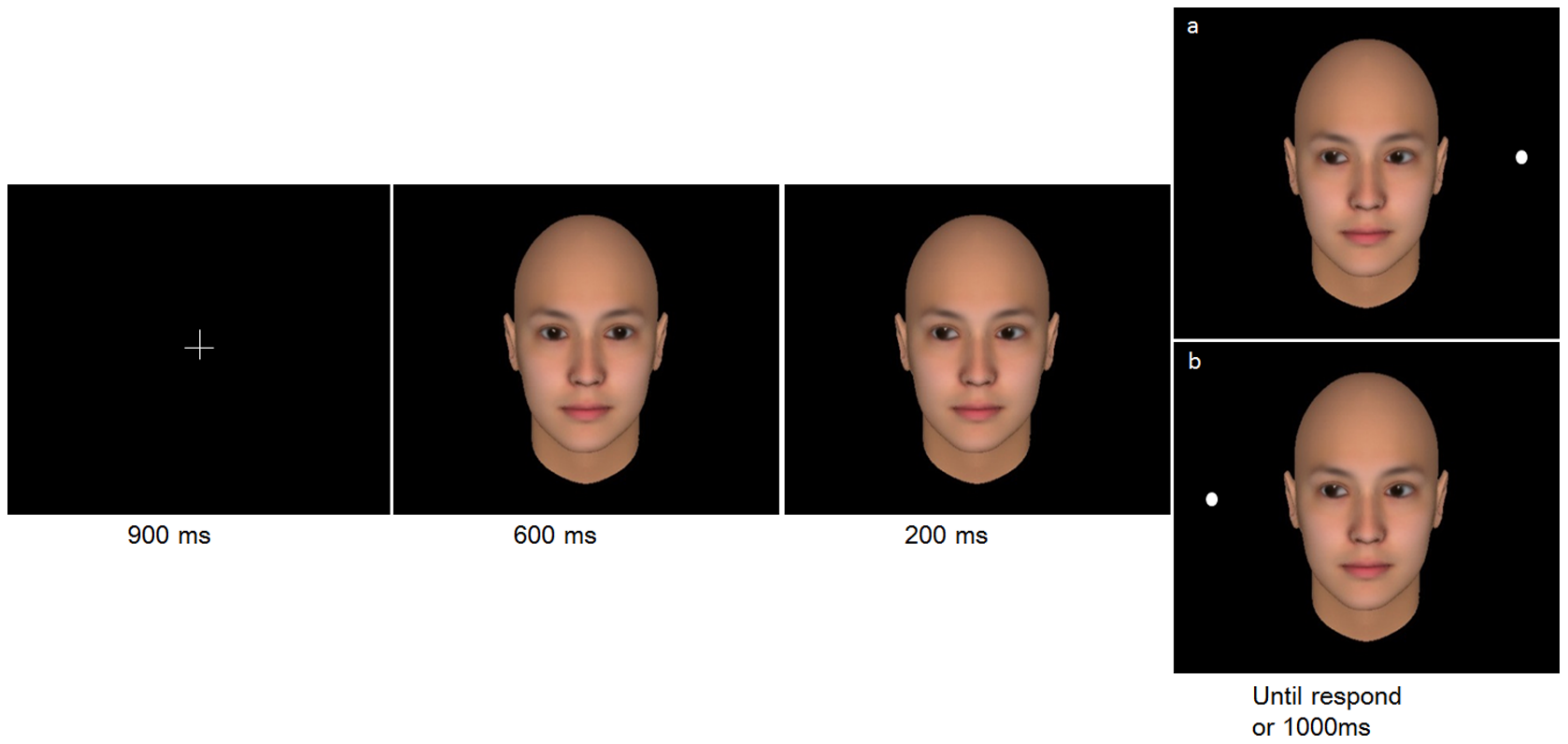
Illustration for the gaze cueing task: (a) the incongruent condition, where the target dot appears in the opposite direction of the gaze cue; (b) the congruent condition, where the target dot appears in the same direction of the gaze cue.

Participants were instructed to press the “F” (left) or the “J” (right) button on the keyboard with the symmetrical fingers of their two hands, to indicate the location of the target, as accurately and quickly as possible. Their response time (RT) was recorded and the gaze cueing effect indicated by the difference in the mean average RT between the congruent and incongruent trials. For each wrong response or when participants failed to respond within 1000 ms, a warning feedback would appear at the center of the screen for 1000 ms (Figure 1) before a new trial was prompted (in the practice trials, feedback was provided for both right and wrong responses).

In addition, to prevent participants from relying on explicit cues, they were told before the task that the location of the target was unrelated to the gaze direction. The locations of the target and the gaze direction were also fully randomized in each block.

After performing the two tasks, participants were fully debriefed, paid, and dismissed. Based on information obtained during the debriefing process, no participants realized that the priming task and the gaze cueing task were related.

### Experiment 2

#### Participants

For this experiment, the participants were 160 undergraduate students of Peking University (80 men, 80 women; Mean age  = 21.54 years, SD = 2.44 years), who received monetary compensation for their time.

#### Ethics statement

As in Experiment 1, the ethics review committee of the Department of Psychology, Peking University approved the protocol details of Experiment 2. Participants provided written consent before taking part in this experiment and were fully debriefed afterwards.

#### Materials

The materials for the gaze cueing task were the same as in Experiment 1.

#### Procedure

In Experiment 2, participants were also asked to complete a priming task and a gaze cueing task that were ostensibly irrelevant to each other.

In the priming task, participants were asked to imagine in 10 minutes that they were in a situation where they were running away from a mountain during an earthquake (high danger) or hiking and finding their way out of a mountain (low danger), as either the leader of their team (high social power) or as a member (low social power). Each condition had 20 women and 20 men participants. Both of the dangerous contexts were rated in a pretest and found to be equally familiar to the participants and significantly different in their degree of danger and risk. To help the participants imagine the situations, they were shown pictures of earthquakes or mountain hiking; participants were also asked to write details of what they imagined, such as a list of the most important issues of concern to a team leader or a regular team member.

The rest procedure of this experiment was the same as in Experiment 1.

## Results

### Experiment 1

We asked three postgraduate students to independently evaluate whether or not the participants' essays in the priming task were related to social power. The judges' ratings were consistent, and confirmed that participants followed the instruction, except for eight participants (3 men 5 women). Two out of the three judges did not rate the essays wrote by these participants as reflecting social power, therefore these participants' data was excluded from the analyses below.

#### Number of error trials in the gaze cueing task

The percentage of trials in which participants responded incorrectly was 0.77% of all trials. The error number was analyzed with a mixed 2×2×2 ANOVA, with gaze cue congruency (congruent vs. incongruent) as a within-participant factor, participants' gender (women vs. men), and social power (high vs. low) as between-participant factors.

The results revealed significant main effects for gaze cue congruency and social power. Specifically, more error responses were found in the incongruent condition, compared to the congruent condition (*M*s = 0.85, 0.08, respectively), *F*(1,48) = 15.41, *p*<.001, 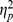  = .243, and for the low social power group, relative to high social power group (*M*s  =  0.67, 0.25, respectively), *F*(1,48) = 5.25, *p* = .026, 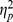  = .099. The interaction between gaze cue congruency and social power was also significant, *F*(1,48) = 4.66, *p* = .036, 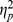  = .089, dominated by the different error response numbers between high and low levels of social power in the incongruent condition (*M*s = 1.27, 0.08, respectively). No other effects, including the main effect or the interaction effects related to gender, were statistically significant (all *F*s<.69).

#### The gaze cueing effect

Trials with error responses or extreme reaction times (beyond 3 standard deviations of participants' mean response time) were excluded from data analysis (accounting for 3.49% of all trials).

We found an overall gaze cueing effect, demonstrated by the participants' longer response times in the incongruent condition (*M* = 361.24 ms), compared to the congruent condition (*M* = 330.48 ms), *t*(51) = 10.36, *p*<.001.

**Figure 2:**
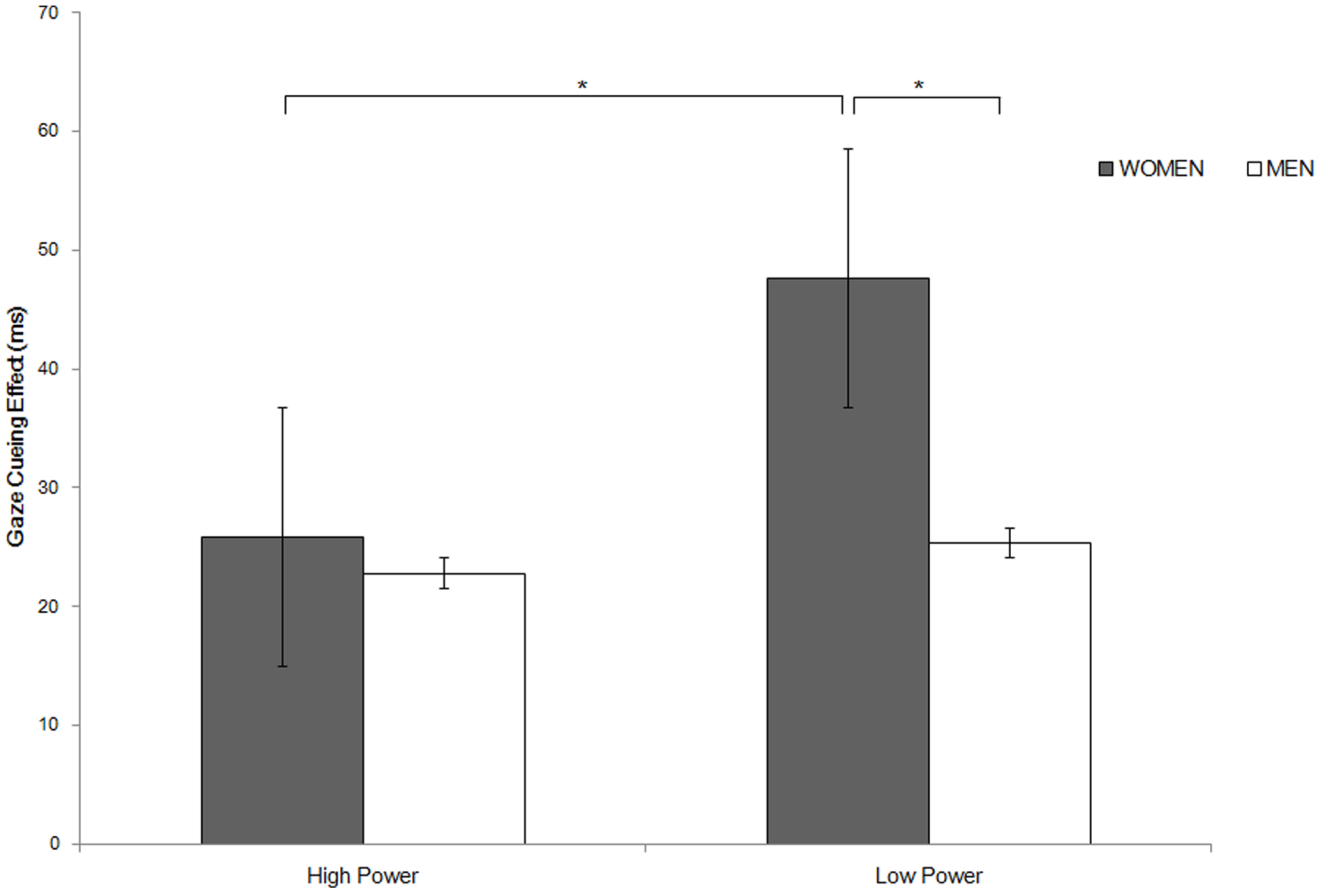
Gaze cueing effects for gender and primed high or low social power in Experiment 1. For this and the following figures, * *p*<.05, ** *p*<.01.

We further conducted a 2×2 ANOVA on the gaze cueing effect (RT incongruent – RT congruent) with participants' gender (men vs. women) and social power (high vs. low) as the between-participant factors. The results showed a significant main effect of social power in that the gaze cueing effect was stronger among participants who had been primed with low social power, compared to those who had been primed with high social power (*M*s = 37.23, 24.29 ms, respectively), *F*(1, 48) = 5.70, *p* = .021, 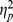  = .106 (Figure 2). The main effect of participants' gender was also significant, *F*(1, 48) = 4.85, *p* = .033, 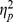  = .092, with a stronger gaze cueing effect found in women, compared to men (*M*s = 36.72, 24.80 ms, respectively). The interaction of the two factors was not significant, *F*(1,48) = 2.69, *p* = .11. However, the planned contrast analysis showed a predicted stronger gaze cueing effect in women than in men, among those who had been primed with low social power, *F*(1,49) = 6.73, *p* = .01, 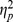  = .121; but not among those who experienced high social power, *F*(1,49) = 0.14, *p* = .71. Meanwhile, as we hypothesized, women primed with high social power exhibited a weaker gaze cueing effect, compared to their low social power counterparts, *F*(1,49) = 7.52, *p* = .009, 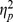  = .133, though this pattern was not observed among men, *F*(1,49) = 0.26, *p* = .613.

### Experiment 2

As in Experiment 1, three postgraduate students independently evaluated the participants' writing in the priming task, and confirmed that all participants followed the instructions in each condition.

#### Number of trials with errors in the gaze cueing task

The total number of trials with wrong responses amounted to 0.82% of all trials. The number of error responses were analyzed with a 2×2×2×2 mixed ANOVA, with gaze cue congruency (congruent vs. incongruent) as the within-participant factor, participants' gender (women vs. men), priming situation (high vs. low danger), and social power (high vs. low social power) as the between-participant factors. The results showed only a significant main effect for gaze cue congruency, *F*(1, 152) = 49.91, *p*<.001, 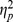  = .247, indicating that more error responses occurred in the incongruent, rather than congruent gaze conditions (*M*s = 0.88, 0.11, respectively).

#### The gaze cueing effect

Trials with error responses or extreme reaction times (beyond 3 standard deviations of participants' mean response time) were excluded from the data analysis, which accounted for 1.98% of all trials.

Like in Experiment 1, the reaction times in the incongruent condition (*M* = 357.18 ms) were longer than those in the congruent condition (*M* = 330.36 ms), *t*(159) = 21.63, *p*<.001, indicating the existence of the gaze cueing effect.

We conducted a 2×2×2 ANOVA on the gaze cueing effect (RT incongruent – RT congruent), with participants' gender (men vs. women), priming situation (high danger vs. low danger), and social power (high vs. low) as the between-participant factors. The results showed a significant interaction between gender and social power, *F*(1,152) = 4.273, *p* = .040, 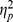  = .027. A simple effect analysis revealed a marginal gender difference in the low social power condition, *F*(1,157) = 3.29, *p* = .071, 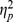  = .021, but not in the high social power condition, *F*(1,157) = 1.20, *p* = .276. Meanwhile, women who were primed with low social power exhibited a marginally stronger gaze cuing effect, compared to women primed with high social power, *F*(1,157) = 3.26, *p* = .073, 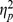  = .020, but such patterns were not significant for men, *F*(1,157) = 1.22, *p* = .271.

**Figure 3:**
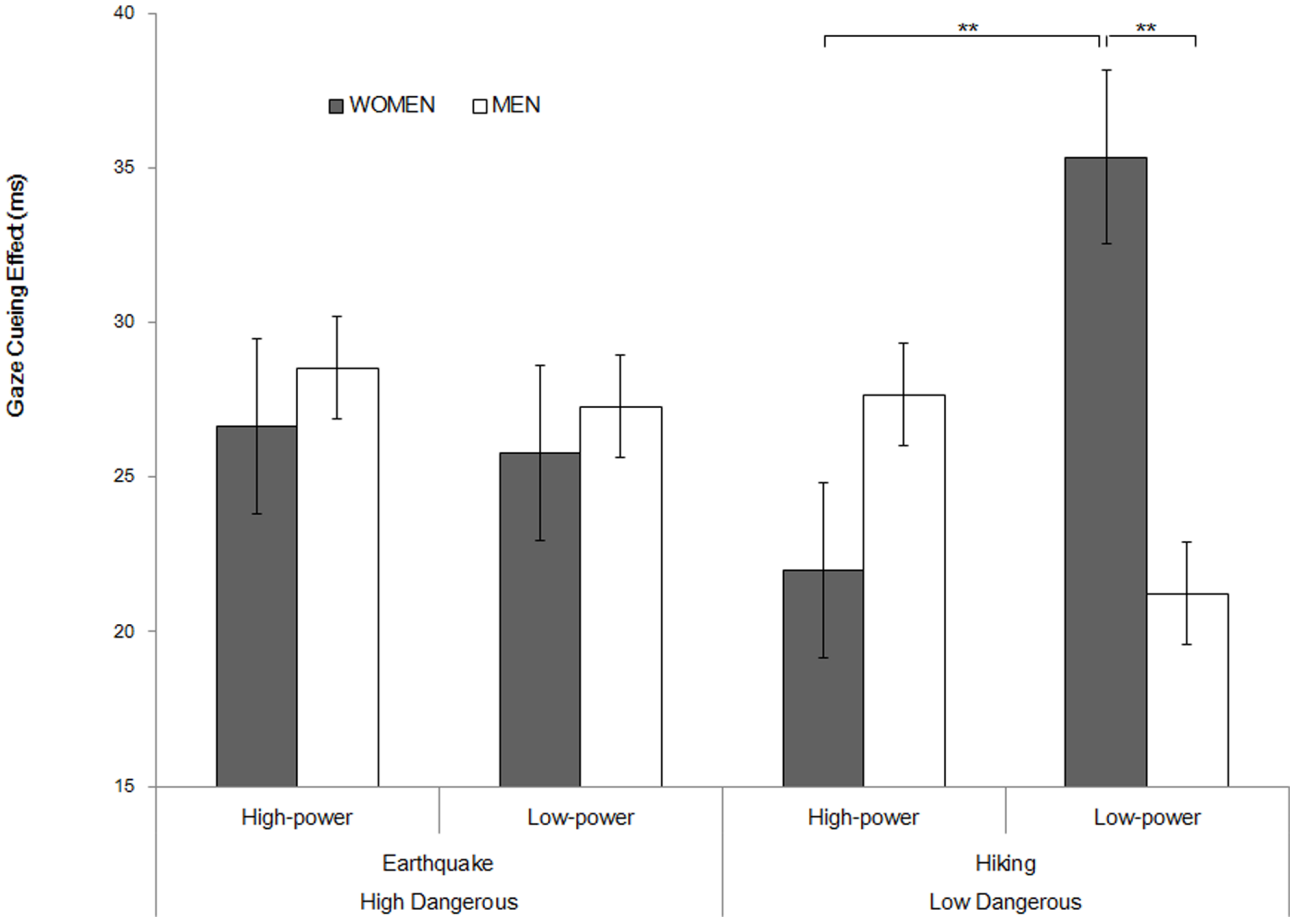
Interaction of social power, gender, and dangerous context in Experiment 2.

Importantly, this two-way interaction was further qualified by the significant three-way interactions of gender, social power, and priming situation, *F*(1,152) = 3.93, *p* = .049, 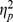  = .025. A simple effect analysis showed a significant interaction between the participants' gender and primed social power in the low danger context (hiking), *F*(2,159) = 8.31, *p* = .004, 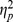  = .050, but not in the high danger context (earthquake), *F*(2,159)<0.01, *p* = .952. Specifically, in the low danger context, women with a low sense of social power exhibited a stronger gaze cueing effect (*M* = 35.37 ms), compared to their male counterparts (*M* = 21.24 ms), *F*(2,159) = 8.52, *p* = .004, 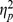  = .051, or to women with a high sense of social power (*M* = 21.98 ms), *F*(2,159) = 7.63, *p* = .006, 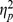  = .046. However, the gender difference disappeared for the high social power condition (*M* = 21.98, 27.68 ms for women and men, respectively), *F*(2,159) = 1.76, *p* = .186. No significantly different gaze cueing effect was found between the men with high and low social power (*M*s = 27.68, 21.24 ms), *F*(2,159) = 1.76, *p* = .186 (Figure 3). No other effects were significant (*p*s>.19).

## Discussion

We adopted different priming methods in two experiments to explore how one's primed social power might affect the subsequent attending to another person's gaze, with a comparison between men and women. In Experiment 2, we also varied the level of danger in the context. In both experiments, the participants demonstrated the gaze cueing effect, even though they were explicitly instructed about the irrelevance of the gaze direction and the target location. This is consistent with previous research showing that gaze following is a reflexive and automatic process [10], [11], [42]. The findings from Experiment 1 also showed a stronger gaze cueing effect among participants who were primed with lower social power, and these participants also made more response errors when judging the location of the target when the gaze cue was incongruent with the location of the target in the gaze cueing task, compared to participants who were primed with high social power. In other words, individuals primed low social power were more easily influenced by the gaze direction of others in distributing their attention. This robust association between the lower power/status (even when generated by a temporary lab priming task) and the sensitivity toward the gaze directions of others may reflect an automatic process that is deeply rooted in the evolving process of social interactions. From an evolutionary perspective, shifting attention to the gaze direction of others is an effective way to detect potential danger or locate food, aiding survival in the environment [43], [44], especially for those with lower social power who may be relatively less independent, compared to those with higher social power [25], [26].

Importantly, as hypothesized in Experiment 1, we found that women primed with lower social power showed a stronger gaze cueing effect, compared to their male counterparts. Nevertheless, women and men who were primed with high social power did not show a significant difference from each other. This same interaction pattern between social power and gender was replicated in Experiment 2.

Research has shown a general stronger gaze cueing effect being exhibited by women, compared to men [40], [41], and the gender difference has been postulated to be due to women's greater sensitivity to social cues [39], [45]. This idea could also explain our findings that a stronger gaze cueing effect is seen in women, compared to men, in the primed low social power condition. In our study, the lack of gender difference in the gaze cueing effect in the high social power condition implies a more general explanation for women's stronger gaze cueing effect, possibly stemming from women's relatively lower position in social hierarchy [37], [38], which could also account for the gender differences for social sensitivity. In the same vein, our findings may reflect different strategies used by women and men in their social interactions. The literature suggests that women generally cooperate more than men [46]. In our study, the primed lower social power may cause women to be more cooperative and show a tendency to be followers. Further research should directly examine whether or not the gaze cueing effect is related to different strategies used by different social groups.

Experiment 2 revealed that the above interaction effect of gender and primed social power on gaze following only existed in the low danger context. When the context was highly dangerous, such as when participants imagined escaping from an earthquake, it seemed to eliminate the (interaction) effect. This modulating effect of the dangerous situation may be understood by analyzing people's possible experiences in these situations. Earthquakes are usually associated with terror and death, and the traumatic events in China's recent past may still be held vividly in memory by Chinese people. On the other hand, while some risk is present in the mountain hiking situation, the low level of danger would likely be associated with adventure, rather than with a life-threatening experience. Thus, imaging a highly dangerous earthquake situation might be closer to a powerful “real” threatening experience, with higher level of anxiety that could overwhelm the participants, in contrast to the imagined hiking task. Although the exact mental processes that the participants went through in the different types of dangerous situations is not the subject of this study, a powerful threatening situation likely causes people to distribute fewer mental resources to their own social factors, such as gender or social power, which could explain the lack of effect of these social factors.

Social hierarchy in human society is a complicated phenomenon. Although social status influences social power [2] and those of higher social status usually possess higher social power, the extent to which social status and social power function in a similar way to influence psychological processes is unclear. In addition, while social status in people's interaction is often relative (i.e., another person's higher status/power might suggest one's own lower status/power), whether or not the relative statuses exert similar effect on human interaction is unexplored. Unlike previous research on social status moderating the gaze cueing effect, which mainly focused on the different status characteristics of faces seen by participants (such as identity [18], [21], [47] or physiognomic traits [19], [36] of faces), our study manipulated the participants' own social status. We demonstrated that individuals change their gaze following behavior even when the status of others is unknown, suggesting that one's perceived social power is important in shaping social attention induced by gaze. We also found that the level of danger in a situation significantly interacts with one's perception of social power as well as gender to modulate the gaze following patterns. Future research should continue to investigate how contextual characteristics and the relative social status affect people's social attention.

In summary, we conducted the first study to demonstrate the sense of social power as a strong factor that affects gaze following behavior. Our finding of a stronger gaze cueing effect among those with perceived lower social power supports the idea of gaze following being evolutionarily adaptive. More importantly, we demonstrated the collective effect of primed social power and other social factors, such as gender and the level of danger in the context. Although the specific mechanisms need further research, our study provides new insights for understanding the role of social power and the fundamental aspects of social interaction.

## Supporting Information

Data S1 **Experimental data for Experiment 1 and 2.**
(XLSX)
